# Polyclonal alpaca antibodies protect against hantavirus pulmonary syndrome in a lethal Syrian hamster model

**DOI:** 10.1038/s41598-021-96884-6

**Published:** 2021-08-31

**Authors:** Patrycja Sroga, Angela Sloan, Bryce M. Warner, Kevin Tierney, Jocelyne Lew, Guodong Liu, Michael Chan, Yvon Deschambault, Derek R. Stein, Geoff Soule, Logan Banadyga, Darryl Falzarano, David Safronetz

**Affiliations:** 1 Department of Medical Microbiology, University of Manitoba, Winnipeg, MB Canada; 2 Zoonotic Diseases and Special Pathogens, National Microbiology Laboratory, Public Health Agency of Canada, Winnipeg, MB Canada; 3 Vaccine and Infectious Disease Organization, University of Saskatchewan, Saskatoon, SK Canada; 4 Department of Veterinary Microbiology, University of Saskatchewan, Saskatoon, SK Canada; 5 Present Address: National Centre for Foreign Animal Diseases, Canadian Food Inspection Agency, Winnipeg, MB Canada; 6 Present Address: Cadham Provincial Laboratory, Winnipeg, MB Canada

**Keywords:** Infectious diseases, Viral infection

## Abstract

The use of antibody-based therapies for the treatment of high consequence viral pathogens has gained interest over the last fifteen years. Here, we sought to evaluate the use of unique camelid-based IgG antibodies to prevent lethal hantavirus pulmonary syndrome (HPS) in Syrian hamsters. Using purified, polyclonal IgG antibodies generated in DNA-immunized alpacas, we demonstrate that post-exposure treatments reduced viral burdens and organ-specific pathology associated with lethal HPS. Antibody treated animals did not exhibit signs of disease and were completely protected. The unique structures and properties, particularly the reduced size, distinct paratope formation and increased solubility of camelid antibodies, in combination with this study support further pre-clinical evaluation of heavy-chain only antibodies for treatment of severe respiratory diseases, including HPS.

## Introduction

Orthohantaviruses (genus *Orthohantavirus*, family *Hantaviridae*) are a large and diverse group of viruses with a global distribution^1,2^. At the most basic level, they are geographically divided into Old World, endemic in Europe and Asia, and New World, which are prevalent in North and South America, viruses. Pathogenic and non-pathogenic viruses exist in both groups, with unique disease characteristics and target organ systems in humans associated with each^3^. Hemorrhagic fever with renal syndrome (HFRS) is associated with Old World viruses while hantavirus pulmonary syndrome (HPS) occurs in the Americas^4^. Annually, greater than 100,000 HFRS suspected or confirmed infections are diagnosed, primarily in Asia, with mortality rates ranging from < 1 to 15%. In contrast, HPS infections have a lower incidence rate with hundreds of infection confirmed each year, though mortality rates are often 30–50%^1,3^. Currently, there are no approved medical countermeasures to treat or prevent HFRS or HPS^5,6^.

Post-infection, individuals exposed to etiological agents of HPS remain asymptomatic for days to weeks. Disease onset is sudden, with general flu-like symptoms, which can rapidly progress to shortness of breath to respiratory distress requiring hospitalization and, often, mechanical ventilation^4^. Death can occur within 12–36 h of hospitalization, leaving only a brief opportunity to treat the disease^7^. Numerous prophylactic vaccination platforms have been evaluated and shown near complete efficacy at preventing infections; however, without improvements in forecasting potential outbreak areas or years, their use would be limited to high-risk individuals^5,6^. Using the Syrian hamster model of disease, direct acting antivirals have shown efficacy in treating infections, however only when treatment is initiated early post-infection, making them less attractive for treating symptomatic human patients^8^. Similar conclusions were made when assessing the efficacy of the broad-spectrum antiviral ribavirin, which in clinical studies showed no positive effect on the outcome of HPS in patents^9,10^.

The recent use of antibody therapies in treating human infections with a multitude of pathogenic viruses including but not limited to Ebola, Lassa, Junin, and Zika viruses, has demonstrated promising results^11,12^. Specific to orthohantaviruses, clinical data support the hypothesis that antibody titer at the time of admission inversely correlates with disease severity and clinical outcome^13^. This means patients admitted with high titer antibody responses are more likely to experience decreased disease manifestations and are more likely to survive, compared to those with low or no detectable antibodies. Thus, passive antibody treatments have also been evaluated in animal models of HPS and HFRS with promising results^8^. These have focused primarily on avian or humanized polyclonal IgG antibodies often generated by DNA vaccination of geese and ducks^14,15^ or transchromosomal bovines (TcB) which have been genetically engineered to produce fully human IgG antibodies^16,17^. Each approach has its own advantages. In general, avian species produce high titer IgY antibodies with greater target specificity and binding avidity than mammalian IgG antibodies which, due to their structure, do not activate human complement and are unable to cause an inflammatory response yet retain all neutralizing capabilities^18^. Antibodies generated from TcB are attractive because they are human IgG antibodies and large volumes of polyclonal sera can be produced in a short period, making this platform ideal in outbreak settings^19^. Recently, the protective ability of neutralizing monotherapy using monoclonal antibodies derived from HPS survivors from South America as well as experimentally produced in mice were also assessed in the hamster model ^20,21^.

Based on the success of these studies, we sought to explore the use of neutralizing antibodies using a non-conventional antibody structure. The unexpected discovery of heavy-chain only IgG antibodies in 1993 was the first example of naturally produced antibodies that differ from the conventional structure^22–24^. Camelids, including llamas, alpacas, and camels, naturally produce heavy-chain IgG antibodies that constitute anywhere from 10 to 60% of total serum IgG antibody^24,25^. As the name implies, heavy-chain antibodies do not possess light chains nor do they contain CH_1_ domains, which typically interact with the light chains in conventional antibodies^22,25^. The camelid heavy chain is approximately 45 kDa in size rather than the conventional 50 kDa size^25,26^. This structural difference is important for the secretion of heavy-chain antibodies, as conventional heavy chains remain within the endoplasmic reticulum until the heavy chain and light chains interact^23,25^.

The main area of focus regarding heavy-chain antibodies is the variable domain (VH), a small single-domain antibody (sdAb) that is 12–15 kDa in size^23,25^. At a fraction of the conventional antibody size, the 12–15 kDa sdAbs possess unique characteristics that contribute to enhanced binding capabilities. The size results in increased solubility and the extended CDR1 and CDR3 loops, compared to conventional VH domains, allow for a unique convex shape with a great amount of flexibility, allowing the antibody to recognize epitopes that are typically inaccessible to conventional paratopes^22,24,25^. Further, owing to their small size, sdAbs are less immunogenic the conventional antibodies which theoretically reduces the risk of adverse reactions, including serum sickness, when they are used as immunotherapies^22,24,27^. For this reason they are actively being investigated as antivenoms^27^. Alpacas produce two types of heavy-chain antibodies, the long-hinged IgG_2_ and the short-hinged IgG_3_^25,28^. Little is known regarding the exact functions of each heavy chain antibody type, however studies have indicated that vaccination results in the production of effective neutralizing IgG_3_ and IgG_1_ antibodies, with IgG_1_ levels slightly dominating because they are more abundantly produced^29^. The production of neutralizing IgG_2_ has been documented in limited amounts and all three IgG sub-types are effectively capable of binding Fc receptors on macrophages^29^.

SdAbs have been evaluated for a few viral pathogens, including respiratory pathogens such as respiratory syncytial virus (RSV) as well as influenza virus and Middle East respiratory syndrome (MERS-CoV), both of which provided protection against lethal viral challenge in appropriate animal models^30,31^. The goal of this study was to generate neutralizing polyclonal serum by DNA vaccination of alpacas and assess its protective efficacy in the lethal Syrian hamster model of hantavirus pulmonary syndrome (HPS).

## Results

### DNA vaccine preparation

The sequence authenticity of the cloned ANDV-GPC gene was verified by nucleotide sequencing which demonstrated no mutations when compared to published sequences (data not shown). ANDV glycoprotein expression was evaluated by standard Western blot analysis on human embryonic kidney 293T (HEK 293T) cells at 72 h post-transfection with pCAGGS-ANDV-GPC. Monoclonal antibodies confirmed expression of Gn at approximately 65–70 kDa and Gc at approximately 50–55 kDa from pCAGGS-ANDV-GPC. Expression was confirmed for each lot of pCAGGS-ANDV-GPC used for vaccinating alpacas.

### Vaccination of alpacas and determination of neutralizing antibody titers

**Figure 1:**
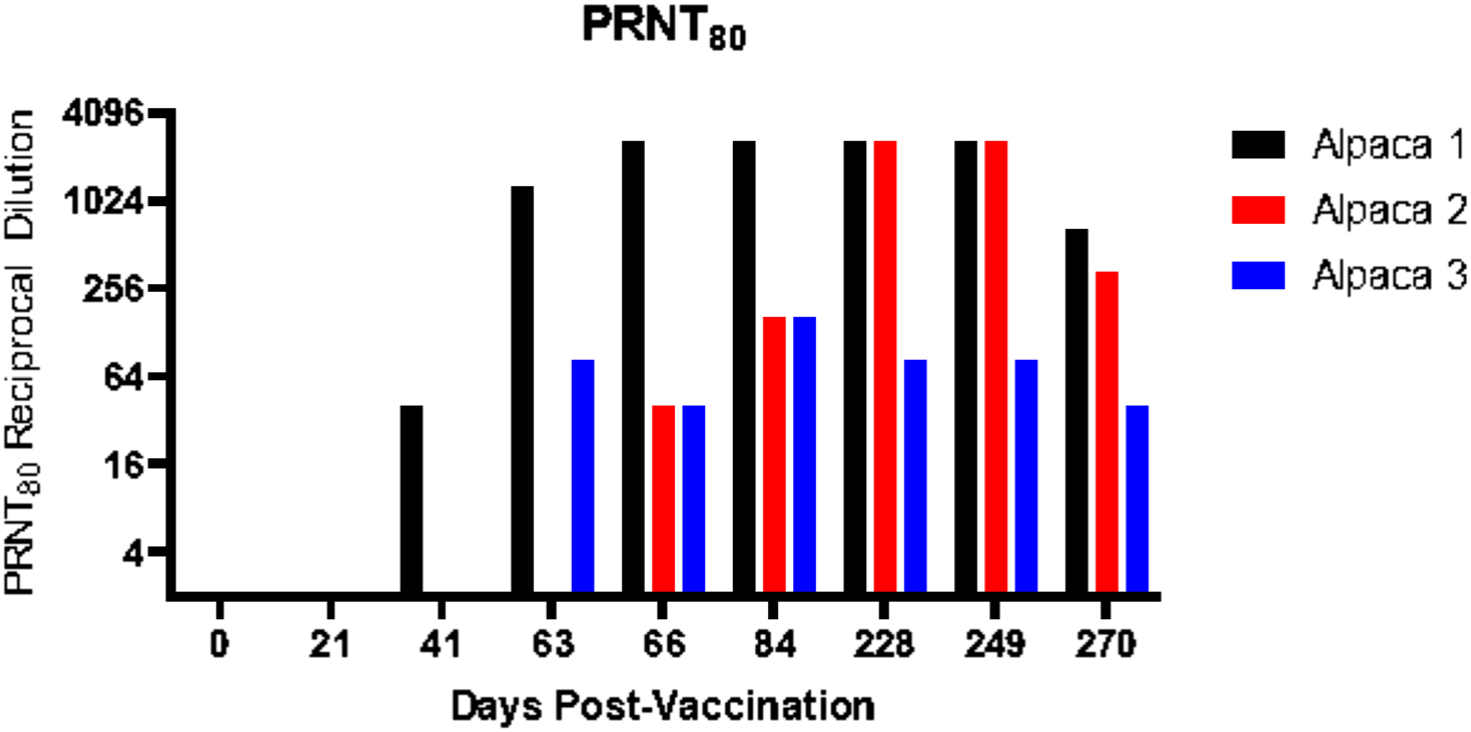
Andes virus neutralizing titers of polyclonal alpaca serum. Serial blood samples were collected from immunized alpacas at the indicated time points and plasma was evaluated using a surrogate VSV-based PRNT assay for neutralization titers.

Three alpacas were immunized via the intradermal route with pCAGGS-ANDV-GPC. Serial blood samples were collected from these animals at the indicated time points, separated into the plasma fraction and examined by a surrogate PRNT assay for neutralization titers (Fig. 1). One animal (referred to herein as alpaca 1) had the earliest and highest response to the vaccinations with PRNT_80_ titers improving from 80 at day 41 to 2560 on days 66 and 84. Titers in this alpaca subsequently decreased during the resting period and although they briefly rebounded to 2560 following the day 228 boost, the final titer was 640 on day 270. Alpacas 2 and 3 responded poorly to the vaccination schedule and only achieved PRNT_80_ titers of 160 on days 66 and 84. Alpaca 3 continued to respond poorly despite switching to intramuscular administration for days 228 and 249 boosts. For alpaca 2, neutralizing antibody titers increased to 2560 at day 249; however, the titer decreased to 320 by day 270.

### Analysis of IgG subtypes from alpaca serum

Post-FPLC elution, gel electrophoresis confirmed a heavy chain band of approximately 45 kDa for IgG3, a heavy chain band of approximately 45 kDa for IgG2, and both an approximately 50 kDa heavy chain band and a 25 kDa light chain band for the conventional IgG1 antibody. Given alpaca 1 yielded the highest titer of Alp-PcIgG-ANDV, subtypes from this animal were analyzed by PRNT_80_ (Table 1). As expected, no neutralizing antibody activity was seen for the day 0 collection (pre-bleed). The first two vaccinations, administered at day 0 and day 21, generated low levels of neutralizing PcIgG. After the 3rd vaccination on day 41, a large response was generated which was seen for the day 63 collection. For day 63, a neutralizing EC_50_ (dose of antibody at 50% of maximum neutralization activity) value of 3.35 µg/ml was observed (Table 1). The final vaccination at day 63 boosted the neutralizing antibody production once more within the animal, as the EC_50_ value for the day 66 collection decreased to 2.73 µg/ml. The EC_50_ value decreased three weeks after the final vaccine dose prior to testing the animals on day 84–3.19 ug/ml.

**Table 1:** Estimated EC_50_ values for purified Alp-PcIgG and IgG subtypes from alpaca 1 plasma collections.

Sample collection date	Antibody subtype	EC_50_ value (µg/ml)
Day 63	PcIgG	3.35
	IgG1	3.67
	IgG2	12.08
	IgG3	2.14
Day 66	PcIgG	2.73
	IgG1	4.78
	IgG2	38.54
	IgG3	0.83
Day 84	PcIgG	3.19
	IgG1	4.94
	IgG2	N/A
	IgG3	1.39

To identify changes in neutralizing activity between alpaca IgG subtypes, plasma at days 63, 66, and 84 was purified from alpaca 1. The response seen for the day 63 collection shows low EC_50_ values for IgG3 (2.14 ug/ml) and IgG1 (3.67 ug/ml) and a high EC_50_ value for IgG2 (12.08 ug/ml) (Table 1). The EC_50_ value decreased for IgG3 at day 66 (0.83 ug/ml) but increased for IgG1 and IgG2. This time point occurred after the animal received the 4th vaccination, at day 63. The EC_50_ value increased for both IgG1 and IgG3 at day 84, however values for IgG2 were not collected due to insufficient concentration after purification.

Determination of neutralization activity for individual alpaca IgG subtypes revealed high EC_50_ values for IgG2 at days 63, 66, and 84. Values for IgG1 and IgG3 remained low for all 3 time points. Notably, the subtype with the lowest EC_50_ value (the highest neutralizing capacity) was IgG3, a heavy-chain antibody.

### Bioavailability of polyclonal alpaca IgG in hamsters

**Figure 2:**
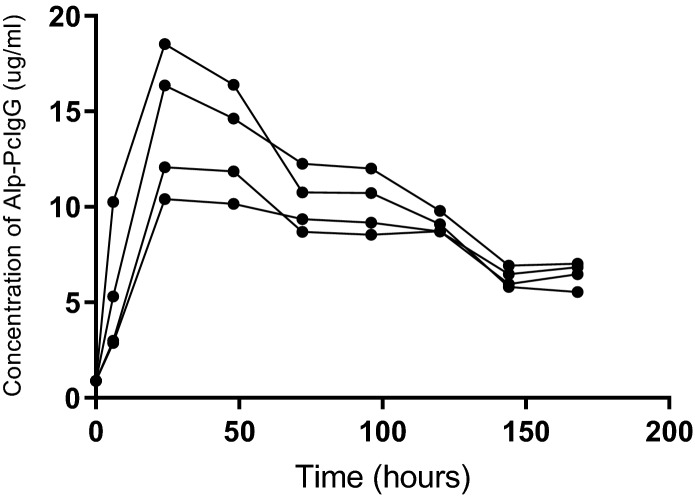
Bioavailability of Alp-PcIgG in Syrian hamsters. Four Syrian hamsters were administered 100 mg/kg polyclonal alpaca IgG via subcutaneous injection and bled at 6, 24, 48, 72, 96, 120, 144, and 168 h post-treatment. Serum Alp-PcIgG levels were assessed by ELISA using a standard curve of naïve PcIgG of known concentration.

Using purified polyclonal antibodies from alpaca’s immunized against ANDV glycoproteins (Alp-PcIgG-ANDV) collected from alpaca 1 at day 270, a single group of four hamsters received 100 mg/kg of Alp-PcIgG-ANDV each. Peak Alp-PcIgG-ANDV detection in each hamster occurred at the 24 h post-treatment time point and ranged from 10 to 18 μg/ml (Fig. 2). After 24 h, the Alp-PcIgG-ANDV concentration decreased, plateauing between 70–100 h, after which it continued to decrease. At 7 days post-administration, between 40 and 60% of the input Alp-PcIgG-ANDV remained detectable in the hamsters.

### Therapeutic evaluation of alpaca PcIgG against Andes virus challenge

**Figure 3:**
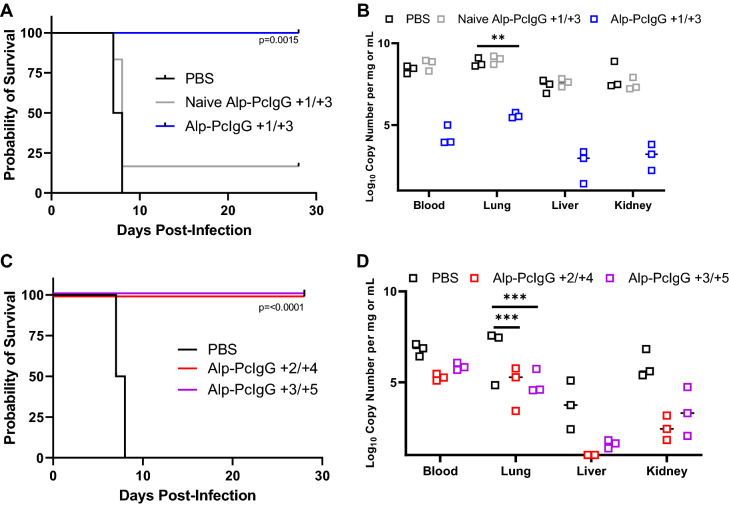
Survival and ANDV RNA levels in Alp-PcIgG-ANDV treated Syrian hamsters. Syrian hamsters were infected with 154 FFU of ANDV and treated with either PBS, purified naïve alpaca IgG, or ANDV-specific Alp-PcIgG at different time points post-infection. (**A**) Survival curve following ANDV infection and indicated treatment on days + 1/ + 3 with respect to ANDV challenge. (**B**) Viral RNA levels in blood and tissues of treated or mock-treated (days + 1/ + 3, with respect to challenge), ANDV infected hamsters. (**C**) Survival of hamsters treated at extended time points (days + 2/ + 4 or days + 3/ + 5, with respect to ANDV challenge) with ANDV-specific Alp-PcIgG or PBS. (**D**) Viral RNA levels in the blood and tissues of treated (days + 2/ + 4 or days + 3/ + 5, with respect to ANDV challenge) and untreated ANDV infected hamsters. For (**A**) and (**C**), n = 6 per group. For B and D, n = 3 per group euthanized on the day post-infection when control animal met the criteria for euthanasia. For (**A**) and (**C**), significance assessed by Log-rank (Mantel-Cox) test. For (**B**) and (**D**), significance was assessed by two-way analysis of variance. ** = *p* < 0.01, *** = *p* < 0.001.

Groups of 9 hamsters (female, 4–6 weeks old) were challenged by intraperitoneal injection of 154 focus-forming units of ANDV Chile-9717869 (representing a dose equal to 100 times the 50% lethal dose). In the first iteration of the study, individual groups were treated on days 1 and 3 post-infection (p.i.) with either purified Alp-PcIgG-ANDV at 100 mg/kg, naïve alpaca polyclonal sera, or PBS by subcutaneous injections. Control (naïve and PBS treated) animals began to demonstrate signs of clinical disease including preference for segregation and breathing abnormalities at day 6 p.i., at which point three hamsters per group were humanely euthanized and samples collected for viral load analysis by RT-qPCR. Between days 7 and 8 p.i., all PBS treated hamsters and 5 of 6 hamsters treated with naïve polyclonal alpaca antibodies developed advanced disease (breathing distress) and were humanely euthanized (Fig. 3A). All six hamsters treated with Alp-PcIgG-ANDV survived challenge with no apparent signs of clinical disease. Consistent with this, RT-qPCR analysis conducted on blood, lung, liver, and kidney collected at day 6 p.i. demonstrated significantly reduced viral levels in specimens from Alp-PcIgG-ANDV treated hamsters compared to controls (Fig. 3B).

**Figure 4:**
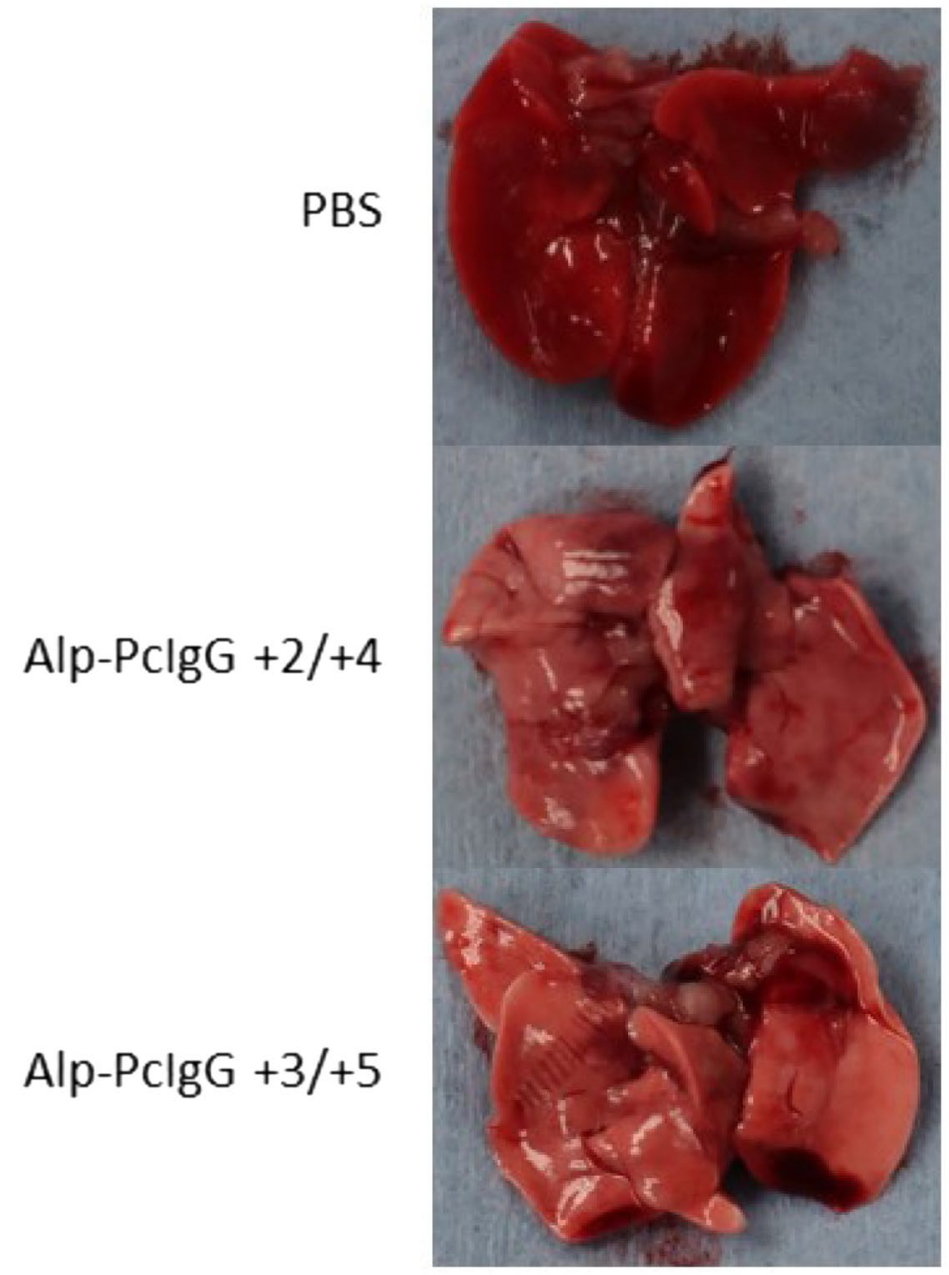
Gross lung pathology in Alp-PcIgG treated, ANDV infected hamsters. Syrian hamsters were infected with 154 FFU of Andes virus (ANDV) and lungs were collected upon euthanasia. Representative lung images for PBS treated hamsters as well as hamsters treated with anti-ANDV polyclonal alpaca IgG (Alp-PcIgG-ANDV) on days + 2/ + 4 and + 3/ + 5 with respect to challenge are shown. Note the diffuse deep red to plum colored appearance of the control (i.e. PBS) treated hamster lungs, indicating substantial inflammation and tissue damage. In comparison, lungs from the Alp-PcIgG treated hamsters appear a healthy pinkish color with rare areas of deeper red.

In a follow up study, Alp-PcIgG-ANDV treatments were given at days 2 and 4 or 3 and 5 p.i., with control hamsters receiving PBS on days 3 and 5. Similar to the first study, all hamsters receiving Alp-PcIgG-ANDV survived lethal challenge with no signs of illness whereas control hamsters developed advanced disease and were euthanized between days 7 and 8 pi (Fig. 3C). Not surprisingly, increased levels of viral RNA were detected in treated animals in specimens collected at day 6 p.i.; however, significant decreases were still obtained in comparison to control animals (Fig. 3D). Further corroborating the survival observed in treated groups, gross pathology of the lungs demonstrated diffuse deep red coloring of control lungs compared to apparently healthy pink colored lungs of treated animals (Fig. 4).

## Discussion

The correlation between disease outcome and antibody titer in HPS patients has been shown clinically and corroborated experimentally in small animal models^8,13^. Based on these observations, clinical trials using convalescent serum collected from HPS survivors in Chile are already underway^32^. However, due to limited supply of convalescent serum and for safety reasons, passive transfer of this nature may not be sustainable. To this end, experimentally produced antibodies, including IgY, TcB human IgG, mAbs, and, here, heavy-chain only antibodies for the treatment of HPS are being explored. The use of sdAb treatments are still in early development, however they have enormous potential in the field due to their unique properties. Because of their elongated CDR regions, sdAbs can access sterically hindered epitopes that are difficult to target with conventional monoclonal antibodies or antibody fragments^33,34^. In addition, the ability to link sdAbs together to create a dimer or trimer is attractive since it allows for the targeting of multiple epitopes^35,36^. Furthermore, the ability to deliver the sdAbs treatments using a nebulizer would facilitate a quick and less invasive treatment approach directly to the target organ^36^. To date, the most successful sdAb therapeutic is the ALX-0171 nanobody™, used to treat RSV, which is currently in Phase 2 clinical trials^36–38^. This trivalent nanobody™ has demonstrated a greater neutralizing capacity when compared to the currently used RSV monovalent antibody treatment, possesses the unique qualities of being easily manufactured in vitro, and can be delivered directly into the lungs through an inhaler/nebulizer^36–38^.

Based on the success of ALX-0171, we sought to explore sdAbs as a treatment option for HPS in the lethal Syrian hamster model of disease. Using DNA vaccines, immunized alpacas generated ANDV specific neutralizing titers sufficient for these concept studies. Although the neutralizing titers were not as high as expected, optimization of the immunization strategy as well as the DNA vaccine is underway and would be expected to improve on these values. Nevertheless, it is important to note that the EC_50_ value for IgG3 was consistently the lowest among the sub-fractionated antibodies (Table 1), indicating these heavy-chain antibodies yielded the greatest neutralizing capacity.

In the lethal challenge model, post-infection treatments with Alp-PcIgG-ANDV were able to protect hamsters from lethal ANDV disease. The use of differing infectious doses and exposure routes of challenge virus complicates the direct comparison of the results of these studies to others using antibody-based therapies. Depending on the challenge dose, hamsters challenged via the intranasal route succumb to infection between days 12 and 20 post-infection, whereas intramuscular challenge is lethal within 10–14 days and intraperitoneal challenge results in death in 8–12 days^39–41^. The specific times to death associated with each challenge route is highly reproducible in both male and female hamsters and is most likely due to differing viral growth kinetics within the host hamsters^8^. Nevertheless, the timing of apparent signs of respiratory disease and eventual distress is a common feature and occurs approximately 48 h preceding death^8^. In the i.p. challenge model, subcutaneous treatments on days + 3 and + 5 pi were capable of reducing viral burden, tissue specific gross pathology, and signs of disease resulting in 100% survival in these studies (Fig. 3). Based on these results, as well as the bioavailability data from the current study which demonstrated 40–60% of input Alp-PcIgG-ANDV antibodies were detectable at 7 days post-administration (Fig. 2), one could anticipate that treatments could be delayed closer to the time of the appearance of signs of disease. Unfortunately, due to short supply of the purified Alp-PcIgG-ANDV, this could not be done in the current study.

Despite the limitations due to low yields of neutralizing antibodies produced in the DNA immunized alpacas, this study demonstrates the utility of these unconventional antibodies as a therapeutic option for HPS. Further studies aimed at optimizing sdAb yields from immunized alpacas need to be undertaken. The variable results post-immunizations observed here is not uncommon when DNA vaccines are utilized in larger mammalian species^42^. The delivery of increased concentrations of DNA or at more time points may not influence the yields of specific antibodies produced; however species-specific promoters, codon-optimization of transgenes, adding adjuvants or using multiple immunogens like a DNA prime followed by protein/peptide boosts, may minimize the probability of low-responders in outbred animals^42^. These strategies need to be explored in order to conduct large-scale studies aimed at elucidating the protective efficacy of the camelid IgG subclasses for the purpose of identifying and creating high affinity recombinant sdAbs for further pre-clinical evaluations. Further experimentation is also required using disease models with more natural routes of infection (intranasal installation) with delivery of sdAbs treatments directly to the lungs via a nebulizer.

## Methods

### Ethics statement

Alpaca immunizations and blood collection protocols were approved by the University of Saskatchewan Animal Care Committee. Experiments involving hamsters were approved by the Animal Care Committee of the Canadian Sciences Center for Human and Animal Health. All animal work was conducted in accordance with the guidelines and regulations of the Canadian Council on Animal Care (CCAC) in CCAC approved facilities. This study is reported in accordance with ARRIVE guidelines.

All manipulations were conducted by trained staff. Animals were acclimated for at least one week prior to experimental manipulations. All infectious work with Andes virus (ANDV) was performed under BSL-4 conditions at the National Microbiology Laboratory in Winnipeg, Manitoba, Canada. The animals were given food and water ad libitum and monitored daily throughout the course of the experiments.

### Vaccine design and preparation

The glycoprotein precursor coding region of ANDV Chile 9717869 (accession number AF291703)^7^ was cloned into the expression plasmid pCAGGS using standard molecular cloning techniques. Following sequence confirmation, protein expression was confirmed from pCAGGS-ANDV-GPC by Western blot using monoclonal antibodies targeting ANDV glycoproteins (G_N_ and G_C_, AMSBIO LLC). Plasmid purifications were conducted using endo-free extraction columns (Qiagen).

### Generation of anti-ANDV antibodies in alpacas

Three outbred male alpacas (*Vicugna pacos*), roughly 80 kg in size, were procured from a local supplier and housed at the Vaccine and Infectious Disease Organization (VIDO) in Saskatoon, Saskatchewan, Canada. Alpacas were immunized with pCAGGS-ANDV-GPC via intradermal injections. Briefly, 0.5 mg of expression plasmid was mixed with 160 µl of Invivo-Jet PEI transfection reagent (Polyplus Transfection) without an adjuvant and delivered into 8 sites (approximately 250 µl per site) in the neck at 3 week intervals (immunization dose was 1 mg per time point; total immunogen delivered was 4 mg pCAGGS-ANDV-GPC). Blood samples were collected on days 0 (100 ml), 21, 42, 63 (10 ml per time point), 66 (100 ml), and 84 (10 ml) and separated into plasma fractions for determination of neutralizing antibody titers. Based on these results, blood samples were collected immediately prior to further boosts at days 228 (100 ml blood draw) and 249 (100 ml blood draw) post-initial immunization. Boosts were conducted as outlined above with the exception that the single low-responding animal was boosted via intramuscular injections. A final blood draw (100 ml) occurred at day 270 for purification and subsequent animal studies.

### Purification and concentration of alpaca IgG antibodies

Using FPLC it is possible to fractionate polyclonal alpaca sera into IgG1, IgG2 (long hinge), and IgG3 (short hinge) heavy- chain antibodies. All alpaca antibodies bind Protein A, while IgG1 and IgG3 bind to Protein A and G^28,43,44^. The AKTA Pure Protein Purification system with a HiTrap Protein A column (both from GE Healthcare) was used to collect polyclonal IgG antibodies (hereafter referred to as Alp-PcIgG-ANDV). Individual IgG subtypes IgG1, IgG2, and IgG3 were collected using both HiTrap Protein G and Protein A columns (GE Healthcare). Briefly, columns were washed with 20 mM sodium phosphate (pH 7.4) and plasma was applied. Following a column wash, polyclonal IgG was eluted using 0.1 M glycine–HCl (pH 2.7). For isolation of individual subtypes, IgG3 was eluted initially from the Protein G columns using IgG3 elution buffer (0.15 M NaCl, 0.58% acetic acid; pH 3.5), followed by IgG1 using IgG1 elution buffer (0.1 M glycine–HCl; pH 2.7). Flow-through was collected and applied to the HiTrap Protein A column to isolate IgG2 using IgG2 elution buffer (0.15 M NaCl, 0.58% acetic acid; pH 4.5). Eluted antibodies were concentrated using the PierceTM Protein Concentrator PES 10 K MWCO (Fisher Scientific) and dialyzed in 1X GibcoTM PBS (pH 7.2; Fisher Scientific). Concentrations of IgG antibodies were measured using the NanodropTM One Microvolume UV–Vis Spectrophotometer (Fisher Scientific). The presence and purity of the individual subtypes were visually analyzed using gel electrophoresis.

### Bioavailability study

Four female Syrian hamsters (*Mesocricetus auratus*), aged 4–6 weeks, were used in a preliminary bioavailability study of the purified alpaca antibodies. Pre-treatment blood was collected 7 days prior to the study, after which each hamster received 100 mg/kg of Alp-PcIgG-ANDV subcutaneously. Blood samples (90–180 µl) were collected from each hamster at 6, 24, 48, 72, 96, 120, 144, and 168 h post-treatment via the saphenous vein and serum was tested for the presence of alpaca antibodies by ELISA methodologies outlined below.

### Post-exposure antibody protection experiments

The assessment of post-exposure efficacy of the alpaca antibodies in preventing onset of lethal HPS in the hamster model was conducted in two iterations. In the first experiment, 27 Syrian hamsters were randomly divided into three groups of nine animals and infected by intraperitoneal injection with 100 times the 50% lethal dose (LD_50_), representing a challenge of 154 focus forming units (FFU) of ANDV Chile 9717869^45,46^. On days 1 and 3 post challenge, hamsters were treated with either 400 µl of sterile PBS, naïve alpaca PcIgG (purified from pre-immunization blood samples collected from alpacas), or 100 mg/kg of neutralizing Alp-PcIgG-ANDV. When control (i.e. PBS treated) hamsters began demonstrating signs of respiratory illness (on day 6 post-challenge), three hamsters from each group were exsanguinated and lung, liver, and kidney collected to assess viral load. The remaining six animals per group were monitored daily for signs of disease until the end of the study, day 28 post-challenge.

In the second iteration of the study, 27 hamsters were again randomly divided into three groups and infected as above with ANDV Chile-9717869. Alp-PcIgG-ANDV treatments were administered as above except on days + 2 and 4 or + 3 and 5 with respect to challenge. A control group received PBS only on days + 3/5. The remaining procedures, including disease monitoring, euthanasia for sample collection, and choosing study end points were performed exactly as in experiment 1.

### Enzyme-linked immunosorbent assays

For the analysis of hamster sera collected during the bioavailability study, an ELISA-based methodology was used to compare timed sample collections to a standard curve derived from pre-bleed samples. Unpooled hamster sera from each collection point were diluted 1:100 in PBS, added to duplicate wells of a Costar 96-well flat bottom plate (Corning), and incubated at 4 °C overnight. A standard curve was prepared using naïve hamster serum diluted 1:100 in PBS and spiked with purified naïve polyclonal alpaca IgG (44.4 mg ml) in a two-fold dilution series starting at 1:10,000. Standard curve samples were also added in duplicate to 96-well plates and incubated at 4 °C overnight. The following day, wells were washed 4 times with PBS supplemented with 0.1% Tween 20 and blocked overnight with PBS supplemented with 5% skim milk. The next day, the wells were again washed, incubated with a goat anti-llama IgG (H + L) HRP-conjugated secondary antibody (1:5000 dilution; Fisher Scientific) at room temperature for 1 h, washed, and substrate added. After developing for 30 min in the dark, absorbance was measured at 405 nm. Concentrations of antibodies present in specific samples were determined using the standard curve.

### Neutralization antibody tests

Neutralizing antibody titers in alpaca immune serum were analyzed by plaque reduction neutralizing tests (PRNT) using VSV∆G-ANDV-GPC as a surrogate for authentic ANDV. Plasma samples collected from alpacas were heat-inactivated at 56 °C for 30 min. Two-fold dilutions (1:20 to 1:20,480) of plasma were mixed 1:1 with approximately 50 plaque forming units (pfu) of a recombinant, replication-competent, chimeric vesicular-stomatitis virus (VSV) expressing the ANDV glycoproteins in place of the VSV glycoprotein (VSV∆G-ANDV-GPC), incubated for 1 h at 37 °C, and used to infect confluent monolayers of Vero E6 cells in triplicate in 12-well plates. After a 1 h incubation at 37 °C, inoculum was replaced with 1 ml of overlay supplemented with 2% low melt agarose, incubated for 3 days at 37 °C, and stained with crystal violet overnight. The following day, the crystal violet and agarose were removed from each well, plaques were enumerated, and the PRNT_80_ titer was determined. The same procedure was used for purified alpaca polyclonal IgG or IgG subtypes with the exception of not heat-inactivating the purified samples. Archived hyper-immune hamster sera was used as a positive control for PRNT_80_s.

### Detection of viral RNA

Organ-specific RNA was extracted using Qiagen RNeasy minikits according to manufacturer’s guidelines. Genomic equivalents of ANDV S segment RNA were determined as previously described^47^.
